# Biomarkers and Functional Assays of Epithelial Barrier Disruption and Gastrointestinal Dysmotility in Critical Illness—A Narrative Review

**DOI:** 10.3390/nu15184052

**Published:** 2023-09-19

**Authors:** Julianna Martinez, K. Marco Rodriguez Hovnanian, Enid E. Martinez

**Affiliations:** 1 Rutgers, Robert Wood Johnson Medical School, New Brunswick, NJ 08901, USA; jlm713@rwjms.rutgers.edu; 2 Mucosal Immunology and Biology Research Center, Massachusetts General Hospital, Boston, MA 02129, USA; krodriguezhovnanian@mgh.harvard.edu; 3 Department of Anesthesiology, Critical Care and Pain Medicine, Boston Children’s Hospital, Boston, MA 02115, USA

**Keywords:** gastrointestinal function, epithelial barrier, gastrointestinal motility, critical care

## Abstract

Enteral nutrition in critically ill children has been associated with improved clinical outcomes. Gastrointestinal dysfunction often impedes the timely initiation and advancement of enteral nutrition and can contribute to immune dysregulation and systemic inflammation. Therefore, assessing gastrointestinal function, at a cellular and functional level, is important to provide optimal enteral nutrition therapy and reduce the gastrointestinal tract’s contribution to the inflammatory cascade of critical illness. In this narrative review, we present an overview of biomarker and functional assays for gastrointestinal dysfunction, including epithelial barrier disruption and gastrointestinal dysmotility, that have been considered for critically ill patients.

## 1. Introduction

Nutrition therapy is part of the care of critically ill children. Delivering a minimum of 60% of a patient’s caloric requirement within the first week of admission to the intensive care unit (ICU) has been associated with improved clinical outcomes [1]. Nutrition can be delivered via the enteral or parenteral route. The enteral route is preferred as it promotes gut homeostasis, is more physiologic, and can be provided early in a patient’s clinical course in the ICU, unlike parenteral nutrition [2,3]. However, enteral nutrition delivery is often limited by gastrointestinal dysfunction in critical illness. The pathophysiology of gastrointestinal dysfunction in critical illness is complex and has been previously reviewed [4,5,6]. Gastrointestinal dysfunction includes epithelial barrier disruption and gastrointestinal dysmotility, which can affect nutrient digestion and absorption, and increase the risk for systemic inflammation and bacterial translocation [4,7]. Gastrointestinal dysfunction affects between 40–80% of critically ill children and has been associated with a longer length of stay, mortality, and enteral nutrition intolerance [8,9,10]. This wide range of potentially affected children is due to the diverse definitions used to identify gastrointestinal dysfunction, from biomarkers to bedside clinical assessments. Defining gastrointestinal dysfunction accurately, in a timely manner, and with widely applicable methods is key to providing optimal enteral nutrition in the ICU.

In this narrative review, we present an overview of the varied approaches published to date to identify gastrointestinal dysfunction in critical illness divided by those that determine epithelial barrier disruption and those that determine gastrointestinal dysmotility. We performed a literature search in PubMed including the following terms, [(gastrointestinal OR gut OR gastric) AND (biomarker OR diagnosis OR symptoms) AND (motility OR epithelial barrier OR gut leak) AND ((critical AND care) OR (critically AND ill) OR (intensive AND care))]. Manuscripts focused on gastrointestinal dysfunction in critical illness, including all study designs, were reviewed for inclusion. Additional manuscripts pertinent to the topic of interest either previously identified by the authors or identified through the review of the described literature search, were also considered.

## 2. Epithelial Barrier

A healthy and intact epithelial barrier is needed for normal gastrointestinal function and homeostasis. The gastrointestinal epithelial barrier plays a key role in nutrition digestion and absorption, as well as immune regulation, which carefully balances the passage of key nutrient building blocks and healthy immune signaling proteins while simultaneously blocking toxins, bacteria, or other metabolites that may cause direct or indirect injury to the GI tract [11]. Cellular signaling from epithelial cells and other cells in the epithelial barrier, such as the enteroendocrine cells, also regulate motility. A healthy epithelial barrier depends on the sustained repopulation of diverse gastrointestinal cells including epithelial cells, enteroendocrine cells, and goblet cells, and the complex and carefully regulated tight junctions that keep the barrier between these cells intact. Therefore, the status of the epithelial barrier may be reflected by markers of cellular health, markers of tight junction integrity, and functional testing. Table 1 summarizes markers for gastrointestinal epithelial barrier health. Multiple aspects of critical illness like sepsis and acute respiratory distress syndrome, render the gastrointestinal epithelial barrier dysfunctional, therefore the ability to assess the health of the epithelial barrier is important in critical illness [12]. The consequences of a disrupted epithelial barrier include inadequate nutrient absorption, systemic inflammation, and bacterial translocation, which has been reported in anywhere up to a quarter of certain pediatric critically ill populations such as those who have undergone cardiopulmonary bypass for congenital heart disease surgery [13].

### 2.1. Markers of Cellular Health

The most commonly examined markers of epithelial barrier cellular health have been citrulline and intestinal-fatty acid binding protein (IFABP). Citrulline is a marker of cell mass. Low serum levels of citrulline have been detected in studies of patients with enteric atrophy such as short gut syndrome [14]. Intestinal-fatty acid binding protein is an intracellular protein in gastrointestinal epithelial cells that can be measured in circulation when there has been cell injury or cell death [15]. Therefore, low citrulline levels reflect low cellular mass and high IFABP levels reflect cellular death. Citrulline and IFABP have been examined in children with congenital heart disease, who have undergone surgery and required cardiopulmonary bypass [10]. Citrulline levels were noted to decrease and IFABP levels to increase post-cardiopulmonary bypass in this cohort [10]. Intestinal-fatty acid binding protein in serum and urine, were also noted to be elevated in a cohort of neonates with surgical abdominal pathology who developed necrotizing enterocolitis but not those with sepsis, reflecting tissue-specific changes [16]. The use of urinary samples in this study also serves as a promising non-invasive method of assessing gut health, which is pertinent in pediatric patients for whom blood draws may be limited by patient weight [16]. In critically ill adults, elevated IFABP and low citrulline were associated with greater severity of illness based on inflammatory markers, markers of end-organ function, and greater use of vasoactive agents and antibiotics [17]. In this adult cohort, elevated sequential organ failure assessment (SOFA) scores, elevated IFABP, and low citrulline levels were associated with greater mortality [17]. In multi-trauma adult patients requiring critical care for more than 5 days, IFABP and citrulline were both found to be elevated the first two days of ICU admission [18]. These levels correlated with length of stay, inflammatory markers, and severity of illness based on the Acute Physiology and Chronic Health Evaluation (APACHE II) and SOFA scores. Intestinal-fatty acid binding protein has also been found to be elevated in patients with sepsis and mechanical ventilation and associated with fluid overload and inflammation [19,20].

### 2.2. Tight Junction Markers

Markers of loss of epithelial barrier integrity have been studied in the context of acute inflammation. Tight junction proteins are a complex set of transmembrane and intracellular proteins that regulate epithelial barrier integrity. Claudins are the largest family of transmembrane proteins and these can be pore-forming or barrier-forming [21,22]. Additional transmembrane proteins are occludin, tricellulin, and Junctional Adhesion Molecules (JAMs) [22]. Intracellular proteins support the scaffolding necessary to maintain the epithelial barrier and include zonula occludens and zonulin [23]. These proteins with diverse functions support or inhibit barrier integrity and have been examined in the context of acute inflammation. In the same study of patients with multi-trauma that considered IFABP and citrulline levels, occludin, claudin-1, tricellulin, JAM-1, and zonulin, were measured in the blood. All of these tight junction proteins, except for JAM-1, were increased in the first two days of ICU admission [18]. In the study of critically ill children who had undergone cardiac surgery, claudin 3, a pore-forming tight junction protein, was increased within the first days after cardiopulmonary bypass [10]. Zonulin, a unique protein that can reversibly disassemble the tight junction complex, has been shown to be elevated in critically ill adults with sepsis, and those requiring mechanical ventilation [20,24]. In children who underwent complex surgery and required care in the ICU, a post-operative pro-inflammatory response was identified, and zonulin, as pre-haptoglobin 2, was found to correlate with the degree of post-operative dysmotility [25,26]. This same relationship between inflammation, zonulin as pre-haptoglobin 2, and gastric dysmotility was replicated in a mouse model [26]. Measurement of tight junction proteins in circulation is limited by their presence in other epithelial and endothelial cell layers such as in the lung, vasculature, and blood-brain barrier, thereby reducing their specificity for gastrointestinal injury. Tight junction proteins also have redundant functions and while some protein levels may be decreased others may be upregulated as a compensating mechanism for epithelial barrier injury or leak. Therefore, the levels of a single tight junction protein may not reflect the functional status of the epithelial barrier, instead, a complement of multiple proteins and functional assays are likely necessary.

### 2.3. Indirect Markers of Epithelial Barrier Health

The loss of epithelial barrier integrity can also be assessed by indirect markers. Microbial components such as lipopolysaccharide (LPS), flagellin, and glucan can be measured in circulation when there is a significant disruption in the epithelial barrier. Lipopolysaccharide binding protein (LBP) and soluble CD14 are acute phase reactants that increase in circulation in response to LPS exposure [27]. Few studies have examined these indirect markers of gastrointestinal epithelial barrier integrity in critical illness, but they have been explored in other patient cohorts with risk for acute inflammation. Flagellin and LPS were shown to be increased in the serum of parenteral nutrition-dependent adult patients with short bowel syndrome who were in stable health, yet undetectable in healthy controls [28]. Glucan and LBP, among other markers for loss of epithelial barrier integrity, were increased in adult patients with severe COVID-19 [29]. Similarly, elevated levels of LBP and zonulin were shown to be increased in patients with multisystem inflammatory syndrome in children (MIS-C) [30]. LBP has also been shown to be elevated in children undergoing complex surgery and requiring ICU care, specifically, LBP was noted to be increased 7-fold post-operatively [25]. The risk for bacteremia or other sources for translocation may limit the specificity of these tests as markers of epithelial barrier health.

### 2.4. Functional Assays of Epithelial Barrier Health

Functional assays for the epithelial barrier such as the double sugar test have been studied in critically ill patients. This test relies on the ability to administer an enteral dose of a known mixture of two non-metabolized sugars and their subsequent measurement in the urine for 5 h after administration. Specifically, one sugar is known to be taken up via the transcellular pathway (e.g., mannitol) and the second is not supposed to cross the epithelial barrier under healthy conditions (e.g., lactulose). An elevated ratio of the second sugar to the first would reflect a loss of the epithelial barrier integrity.

In a cohort of pediatric cardiac surgery patients, the lactulose to mannitol ratio was consistent with increased epithelial barrier leak after cardiopulmonary bypass [10]. This test was also performed in critically ill adults and compared to a healthy cohort. They found that all critically ill patients had an elevated lactulose to mannitol ratio compared to the controls but that there was no relationship between the degree of epithelial barrier leak by this test and severity or etiology of illness [31]. A separate study in critically ill adults identified an association between the degree of epithelial barrier leak, using the double sugar test, and multiorgan dysfunction on ICU admission and after [32]. These studies show that this assay can be performed in critically ill patients. The assay, however, requires the patient to tolerate an enteral dose of the sugar solution, five hours of urine collection, and no renal dysfunction, all of which can limit the universal use of this study as a clinical tool in the ICU. A recent study in children with enteropathy suggested that a 2-h urine collection may suffice [33]. This shortened assay should be studied in critically ill populations, as it may allow for a wider use of the double sugar test in the ICU.

### 2.5. Markers of Epithelial Barrier Health and Enteral Nutrition

Many of the aforementioned epithelial barrier markers and functional assays have been associated with clinical outcomes, but their use in monitoring enteral nutrition tolerance and delivery has not yielded consistent results. Low levels of IFABP and high levels of claudin 3 were associated with a feeding intolerance score in the study of pediatric congenital heart disease patients [10]. In a randomized control study of critically ill adults, initiation of enteral nutrition 24 h after admission was associated with a higher lactulose-mannitol ratio than in patients who started enteral nutrition 6 h after admission, which suggests greater epithelial barrier leak in patients who started nutrition later [34]. Enteral nutrition itself promotes gut homeostasis and therefore early initiation of enteral nutrition may support the preservation of intact epithelial barriers, but whether lactulose-mannitol testing could help predict if a patient will tolerate enteral nutrition remains unanswered by this study. A recent study of critically ill adults examined citrulline and IFABP levels in patients who started enteral nutrition early, defined as within 2 days of ICU admission, versus late, and those that achieved 80% or greater of goal versus not. They found overall increasing levels of citrulline over the first week of ICU admission in patients who started enteral nutrition early, but no relationship between citrulline levels and achievement of 80% of the enteral nutrition goal [35]. Intestinal fatty acid binding protein levels were elevated on day 5 of ICU admission in patients who started enteral nutrition early, and on day 3 of patients who achieved 80% of the enteral nutrition goal [35]. The results between citrulline and IFABP are conflicting, again limiting the ability of these markers to guide the initiation of enteral nutrition or monitor enteral nutrition tolerance. The complexity and variability of results to date don’t allow for the development of an accurate and universally applicable guideline on how to implement or interpret these markers in the ICU setting. However, it is clear that epithelial barrier health should be considered in critical illness not only as it relates to the ability to provide enteral nutrition but also in the context of its potential as a source of inflammation and ongoing critical illness. Therefore, including multiple markers, direct, indirect, and functional assays, should be considered in future studies and in the context of bedside clinical practice.

## 3. Gastrointestinal Motility

Nutrient digestion and absorption from a healthy epithelial barrier rely on the complex coordination of gastrointestinal transit of intraluminal content. In critical illness, however, dysmotility can affect all segments of the GI tract. The esophagus has been shown to be hypercontractile and with high pressure at the gastroesophageal junction resulting in an obstructive physiology [36]. The stomach has delayed emptying and there is a loss of coordination of the antro-duodenal region which can result in backflow from the duodenum. The small and large intestines develop abnormal migrating motor complexes resulting in slow transit as well [7]. In critically ill patients, gastrointestinal dysmotility can result in a higher risk for reflux and aspiration, poor EN tolerance, and abdominal discomfort [8,9]. Techniques such as scintigraphy and manometry are the most comprehensive methods of assessing gastrointestinal transit but they are not universally applicable or available for critically ill patients, particularly pediatric patients. Techniques such as the acetaminophen absorption test and C-octanoate breath test have advanced our understanding of gastric motility in critical illness in the context of research [5,37]. Non-invasive methods and routine bedside assessments of gastrointestinal motility may have greater applicability in the ICU.

### 3.1. Non-Invasive Functional Assays of Gastrointestinal Motility

Electrogastrography (EGG) is a non-invasive technique that measures the electrical activity of the stomach by way of superficial electrodes. Electrogastrography can identify abnormal gastric electrical activity, but it lacks specificity, can be easily affected by artifacts such as intraluminal gas, and may not be performed in patients with abdominal surgery. It has not been studied extensively in the ICU. One study in critically ill adults with septic shock identified abnormal gastric emptying with EGG [38]. They also demonstrated an improvement in gastric emptying after the administration of domperidone in 16% of patients [38]. Given the limitations of EGG, advances in computational analyses have led to the use of high-resolution mapping as an alternative method for measuring gastric electrical activity. The first approach to high-resolution mapping was invasive tissue surface mapping, which has led to growing efforts to translate this technique to non-invasive body surface measurements [39].

Gastric point of care ultrasound (POCUS) is another non-invasive technique with wider potential applicability in the ICU. Gastric US has been studied in healthy pediatric cohorts, the perioperative setting, and in premature and full-term neonates [40]. Assessments of gastric contents, gastric volume, and the antral cross-sectional area have all been considered [40]. Fewer studies have been performed in the pediatric ICU. A recent study in critically ill children compared GRV determined by gastric POCUS to manual decompression of the stomach via nasogastric tube [41]. They identified consistently greater gastric volumes by POCUS than by manual stomach decompression, and 72% met full stomach criteria by the PERLAS score by gastric POCUS performed after manual gastric decompression. [41]. Gastric US can be limited by artifacts from gaseous content, and this affected the acquisition of images in only 10% of patients in this study, supporting the feasibility of this technique in a majority of critically ill children. Gastric volume measured by gastric POCUS has also been correlated to gastric volume measured on MRI and monitoring the gastric antral cross-sectional area over time with POCUS has been utilized to determine gastric emptying [42]. Gastric POCUS has also been studied in the context of enteral nutrition. In a cohort of critically ill adults with sepsis, enteral nutrition was advanced based on the measurement of the gastric antral area by POCUS compared to the measurement of GRV [43,44]. Patients for whom enteral nutrition was guided by gastric POCUS had a shorter time to initiate enteral nutrition and achieve the target and had fewer interruptions [43,44]. Enteral nutrition advancement by gastric POCUS was also associated with improved clinical outcomes including less mechanical ventilation and lower mortality [43,44].

### 3.2. Bedside Assessment of Gastrointestinal Motility

The most common approach to assessing gastrointestinal function is the clinical assessment. There is agreement that clinical findings such as gastrointestinal bleeding, necrotizing enterocolitis, abdominal compartment syndrome, ischemia, or bowel perforation are signs of severe gastrointestinal dysfunction [45]. In patients without severe symptoms, gastrointestinal signs and symptoms such as abdominal distension, abdominal discomfort, absence of bowel sounds, diarrhea, emesis, constipation, and GRV are commonly used to guide enteral nutrition readiness and tolerance [9]. Although intuitively these signs and symptoms should reflect gastrointestinal dysfunction, research confirming their clinical significance is limited. Gastric residual volume measurement is the most commonly reported bedside method of assessing gastrointestinal function, and yet multiple studies have shown its lack of correlation with gastric emptying, enteral nutrition tolerance, or risk for aspiration of gastric content [8,46]. Bowel sounds are a reflection of peristalsis, therefore absent bowel sounds have been widely considered a sign of delayed motility or ileus. However, bowel sounds can be infrequent even during healthy states, such that during a time-limited physical exam their absence would inaccurately diagnose gastrointestinal dysfunction and studies have shown poor concordance among examiners [47]. New technologies record amplified bowel sounds over longer periods of time, reducing the variability among examiners and overcoming the time constraints of a routine physical exam to accurately assess bowel sounds. This technique was applied to 52 patients undergoing chest and neck surgery. They monitored changes in the frequency of bowel sounds over the duration of the surgery noting the expected decrease in bowel sounds secondary to the effects of anesthesia [48]. In a small pilot study, this technique was also applied in adults with septic shock. They found an inverse correlation between inflammation by the measurement of a cytokine (IL-6) and the frequency of bowel sounds [49]. This non-invasive technique could serve as an adjunct to other techniques and biomarkers in understanding changes in gastrointestinal function in critical illness, however further studies are needed to understand how it relates directly to actual gastrointestinal motility and the tolerance of nutrition. For example, studies applying the Enhanced Recovery After Surgery (ERAS) protocol have shown that identifying bowel sounds is not necessary to initiate enteral nutrition [50]. Absent bowel sounds have also not been associated with other signs of gastrointestinal function like passing flatus, having bowel movements, or enteral nutrition tolerance [47].

These non-invasive methods of assessing gastrointestinal dysmotility are limited in that some are still considered research tools, require new equipment and expertise, or have not been sufficiently described in the critically ill population and in relation to nutrition outcomes. A potentially easier and more broadly applicable approach to assessing gastrointestinal function is the use of scores that could integrate parameters associated with both epithelial barrier and motility. A gastrointestinal function score could be applied by multiple bedside providers and allow for the severity of gastrointestinal dysfunction to be categorized. Furthermore, the use of scores deviates away from the focus on single signs or symptoms of gastrointestinal dysfunction, which have been shown to be insufficient to predict clinical or nutritional outcomes [51]. The Acute Gastrointestinal Injury (AGI) score was developed in 2012 by the European Society of Intensive Care Medicine and includes five levels of severity [52]. A recent meta-analysis reported on 14 previous studies using the AGI score. Despite heterogeneity among the studies, they report that 40% of critically ill adults have acute gastrointestinal injury based on this score [53]. They also showed that patients with acute gastrointestinal injury had a higher risk for mortality than those without injury based on the score and that mortality risk was greater as the AGI score increased [53]. This score was recently applied to a pediatric cohort of critically ill patients and found that the majority of patients had no acute gastrointestinal injury but that in those patients who did, the risk for mortality also increased as the AGI score increased [54]. The AGI score is limited by its subjectivity, whereby each score is not anchored in objective signs and symptoms but rather, a subjective interpretation of the degree of effect gastrointestinal dysfunction is having on a patient. Table 2 provides a description of each score and examples of patient findings that could be consistent with a particular score. Recently, a group developed the Gastrointestinal Dysfunction (GID) score based on clinical signs and symptoms, such as absent bowel sounds, emesis, and gastrointestinal bleeding with and without transfusion, and found greater separation for risk of mortality among the most severe scores for dysfunction compared to the AGI score [55]. In a study of critically ill adults, this GID score was correlated to gastric POCUS but not citrulline or IFABP levels [56]. No pediatric specific score has been developed and recent pediatric consensus guidelines could only agree on bowel perforation, ischemia, pneumatosis, and mucosal sloughing as signs of gastrointestinal dysfunction in critically ill children. These signs are clear evidence of gastrointestinal dysfunction but potentially only evident when gastrointestinal pathology is too advanced, therefore limiting the ability to intervene early and prevent life-threatening gastrointestinal dysfunction. [45]. Further research into developing an accurate and clinically relevant pediatric gastrointestinal function score is needed.

### 3.3. Biomarkers of Gastrointestinal Motility

Research advances have highlighted the importance of the microbiome in gastrointestinal health. In critical illness, the microbiome has been consistently shown to be altered, though its relation to clinical outcomes has been varied. Studies have shown a reduction in microbial diversity upon admission to the ICU and an imbalance among species tipping into a pro-inflammatory microbial profile [57]. Such changes have been described in diverse cohorts of patients including blunt trauma, traumatic brain injury, sepsis, and a heterogenous cohort of critically ill children [58,59,60,61]. Studies on probiotics have also had variable results with some suggesting a benefit in ventilator-associated pneumonia, though these results were considered of low quality in a Cochrane review [62]. Importantly, studies to date have not explored how microbiome changes in critical illness are specifically linked to nutrition or gastrointestinal function. Multiple non-ICU studies and translational models have shown the potential effects of diet on the microbiome and are too extensive to summarize here [63,64]. Therefore, it is presumed that nutrition therapies, or lack thereof likely affect microbiome composition. Microbiome analysis as part of a routine clinical assessment for gastrointestinal health in the ICU is not currently possible and its implications are not fully understood, however, it must be recognized that critical illness itself, and many of its accompanying therapies can alter the microbiome and potentially affect gastrointestinal function, short- and long-term. Research to specifically examine the relationship between nutrition, gastrointestinal function, and the microbiome in critical illness is needed.

Gastrointestinal motility is regulated by a complex interplay between the neurologic, immune, and enteroendocrine systems [7]. Biomarkers from these different systems, particularly the enteroendocrine system, may serve as potential diagnostic tools for dysmotility in critical illness. Previous studies have shown associations between gastrointestinal hormones and feeding advancement and tolerance in critically ill patients, including adults and children. Results, however, have been discordant which limits the use of these hormones as clinical markers of gastrointestinal health [65]. Furthermore, similar to markers of epithelial barrier health, it is unlikely that levels of one single hormone will be predictive of gastrointestinal function, and instead an array of various markers will be needed.

## 4. Conclusions

Gastrointestinal function is complex, involving multiple regulatory pathways and redundant systems. Alterations in one aspect of gastrointestinal function do not equate to dysfunction in all systems or consistently result in a clinical phenotype, contributing to the difficulties associated with its diagnosis. However, diagnosing gastrointestinal dysfunction in critical illness, in its varied presentations, is important to understand how it contributes not only to adequate nutrition therapy but also to systemic inflammation and critical illness recovery and prognosis. Future approaches for the diagnosis of gastrointestinal dysfunction are likely to be multimodal, including biomarkers and functional assays. Research is needed to develop multifaceted tools that can be universally applied to critically ill patients.

## Figures and Tables

**Table 1:** Potential Markers of Epithelial Barrier Health in Critical Illness.

Marker	Physiologic Function	Expected Pathophysiologic Finding *
Cellular Health		
Citrulline	Amino acid produced by enterocytes, not integrated into proteins	Epithelial barrier atrophy would be associated with low levels
Intestinal Fatty Acid Binding Protein	Enterocyte intracellular protein that participates in lipid metabolism	Epithelial barrier injury and associated cell death would be associated with high levels
Tight junction proteins		
Claudins	Family of transmembrane proteins; pore- or barrier-forming; present throughout multiple epithelial barriers, some are specific to the intestine	Increase or decrease in levels could be present depending on which claudin is being upregulated
Junctional Adhesion Molecules	Transmembrane protein	Loss of epithelial barrier integrity would be associated with low levels
Zonula occludens	Scaffolding protein	Loss of epithelial barrier integrity would be associated with low levels
Occludin	Transmembrane protein present in multiple epithelial/endothelial barriers	Loss of epithelial barrier integrity would be associated with low levels
Zonulin	Dynamic scaffolding protein that triggers phosphorylation of occludin and its disassembly from the tight junction apparatus	Loss of epithelial barrier would be associated with increased levels
Indirect markers		
Lipopolysaccharide (LPS)	Outer membrane component of gram-negative bacteria	Loss of epithelial barrier integrity would be associated with detection in circulation
LPS-Binding Protein	Acute phase reactant, LPS scavenging protein	Increased in setting of exposure to LPS
Soluble CD14	Soluble glycoprotein serves as co-receptor for LPS	Increased in setting of exposure to LPS
Flagellin	Component of bacterial flagella	Loss of epithelial barrier integrity would be associated with detection

* The described pathophysiologic findings are based on the defined physiologic function of these markers from pre-clinical and experimental research studies. However, clinical studies have not consistently identified these same patterns. Variability in clinical study parameters and limitations associated with how markers are tested, for example, biomarker levels from a serum sample versus a sample from local intestinal tissue, may contribute to the lack of consistency in findings in clinical studies.

**Table 2:** Acute Gastrointestinal Injury Score.

Score	Definition	Potential Clinical Signs and Symptoms
0	No symptoms/No injury	-
1	Self-limiting or transient symptoms from known cause	Post-operative nausea and vomiting, GI dysmotility in septic shock
2	Symptoms requiring treatment or Gl dysfunction impeding nutrition but not affecting the patient systemically	Inability to advance nutrition past “trophic” volume, ileus
3	Persistent severe symptoms or Gl dysfunction not responsive to therapies and/or with systemic symptoms	Evolving intra-abdominal hypertension, persistent enteral nutrition intolerance despite treatment, evolving multiorgan dysfunction syndrome
4	Life-threatening symptoms	Bowel ischemia with necrosis, GI bleed with hemorrhagic shock, abdominal compartment syndrome requiring surgical intervention

Modified from References [52,54].

## Data Availability

This study is a narrative review, and as such has no data available to share.
